# Recurrent malignant ventricular arrhythmias and paresthesia—a mystery revealed as aconitine poisoning: a case report

**DOI:** 10.1186/s13256-023-04304-2

**Published:** 2023-12-21

**Authors:** Ole Christian Mjølstad, Maria Radtke, Eylert Brodtkorb, Frode Edvardsen, Wenche Rødseth Brede, Trond Oskar Aamo, Dag Jacobsen, Mathis Korseberg Stokke, Arne Helland

**Affiliations:** 1 Clinic of Cardiology, St. Olav-Trondheim University Hospital, Torgarden, P.O box 3250, 7006 Trondheim, Norway; 2 Department of Nephrology, St. Olav-Trondheim University Hospital, Trondheim, Norway; 3 Department of Neurology and Clinical Neurophysiology, St. Olav-Trondheim University Hospital, Trondheim, Norway; 4 Department of Clinical Pharmacology, St. Olav-Trondheim University Hospital, Trondheim, Norway; 5 Department of Circulation and Medical Imaging, Norwegian University of Science and Technology, Trondheim, Norway; 6 Department of Clinical and Molecular Medicine, Norwegian University of Science and Technology, Trondheim, Norway; 7 Department of Neuromedicine and Movement Science, Norwegian University of Science and Technology, Trondheim, Norway; 8 Institute for Experimental Medical Research, Oslo University Hospital and University of Oslo, Oslo, Norway; 9 KG Jebsen Centre for Cardiac Research, University of Oslo, Oslo, Norway; 10 Department of Cardiology, Oslo University Hospital Rikshospitalet, Oslo, Norway; 11 Department of Acute Medicine, Oslo University Hospital Ulleval, Oslo, Norway; 12 Institute of Clinical Medicine, University of Oslo, Oslo, Norway; 13 National Poisons Information Centre, Oslo, Norway

**Keywords:** Poisoning, Homicidal poisoning, Aconitine, Diagnostic challenges, Cardiac arrest, Arrhythmias, Epilepsy, Seizures

## Abstract

**Background:**

We report a case of a clinical challenge lasting for 12 months, with severe and unresolved clinical features involving several medical disciplines.

**Case presentation:**

A 53-year-old Caucasian male, who had been previously healthy apart from a moderate renal impairment, was hospitalized 12 times during a 1-year period for a recurrent complex of neurological, cardiovascular, and gastrointestinal symptoms and signs, without any apparent etiology. On two occasions, he suffered a cardiac arrest and was successfully resuscitated. Following the first cardiac arrest, a cardiac defibrillator was inserted. During the 12th admission to our hospital, aconitine poisoning was suspected after a comprehensive multidisciplinary evaluation and confirmed by serum and urine analyses. Later, aconitine was also detected in a hair segment, indicating exposure within the symptomatic period. After the diagnosis was made, no further episodes occurred. His cardiac defibrillator was later removed, and he returned to work. A former diagnosis of epilepsy was also abandoned. Criminal intent was suspected, and his wife was sentenced to 11 years in prison for attempted murder. To make standardized assessments of the probability for aconitine poisoning as the cause of the eleven prior admissions, an “aconitine score” was established. The score is based on neurological, cardiovascular, gastrointestinal, and other clinical features reported in the literature. We also make a case for the use of hair analysis to confirm suspected poisoning cases evaluated after the resolution of clinical features.

**Conclusion:**

This report illustrates the medical challenge raised by cases of covert poisoning. In patients presenting with symptoms and signs from several organ systems without apparent cause, poisoning should always be suspected. To solve such cases, insight into the effects of specific toxic agents is needed. We present an “aconitine score” that may be useful in cases of suspected aconitine poisoning.

## Background

Plant species in the *Aconitum* genus are herbaceous perennials, commonly occurring in the wild in the Northern Hemisphere or grown for ornamental purposes. They contain highly toxic alkaloids, of which aconitine is the most abundant [1]. The toxicity of aconitine is mainly caused by its effect on voltage-dependent ion channels in neural and cardiac tissue, of which sodium channels have been studied the most [2, 3]. The result is impaired neuronal action potential conduction, and a combination of impaired cardiac excitability, conduction defects, and secondary ectopic activation due to dysregulated calcium handling [4].

After oral ingestion, symptoms typically start with perioral paresthesia. Gastrointestinal signs and symptoms follow, such as nausea, vomiting, and abdominal pain. Neuromuscular features include weakness, paresthesia, seizures, and reduced consciousness. Cardiovascular manifestations include severe hypotension, palpitations, brady- or tachycardia, and ventricular arrhythmias. Death is due to cardiac arrest, from either ventricular fibrillation or asystole [2, 5].

In several Asian countries, the use of *Aconitum* plant extracts in traditional herbal remedies is widespread. Poisoning may occur due to inadequate detoxification, normally achieved by boiling, which breaks down aconitine into less toxic compounds [6, 7]. In the Western world, the use of aconitine-containing plants in traditional medicine is rare [8]. However, cases of poisoning occur after misguided self-treatment, for suicidal or homicidal purposes, or as a consequence of plant misidentification [9–12].

We describe a case of multiple surreptitious poisonings, leading to life-threatening arrhythmias and diagnostic confusion before the diagnosis was finally confirmed.

## Case presentation

A previously healthy Caucasian man, now aged in his fifties, was admitted to our university hospital with acute renal (tubular) injury in the fall, 10 and 6 years prior to the year of aconitine poisonings. On both occasions, renal function partly recovered after short-term dialysis. Again, in the fall, 2 years before the aconitine admissions, he presented with severe acute hepatic failure, which recovered spontaneously within a week. Thorough examinations, including renal biopsies, revealed no etiological explanation for these incidents. Poisoning was considered on all three occasions, but no culprit agent was identified. In the same period, he had been diagnosed with a monoclonal gammopathy of undetermined significance (MGUS) with no progression.

During a 12-month period, the patient was admitted to hospital 12 times, presenting with acute and dramatic neurological, cardiovascular, and gastrointestinal features. The first episode occurred nearly 1 year before the correct diagnosis was made. On this occasion, which was proven typical for later episodes, he shortly after a meal felt perioral paresthesias and numbness, along with abdominal pain, nausea, vomiting, and marked dizziness. Family members also reported that the patient had a short loss of consciousness. When examined in the emergency department, he had a severe hypotension of 70/40 mmHg and frequent ventricular ectopic complexes. The clinical examination, including a neurological examination, was otherwise normal. He was observed at the cardiac intensive care unit (ICU) for some hours, during which he fully recovered. He was discharged after 2 days.

During the following months, he had several similar episodes, some leading to hospitalization, while most subsided at home within a few hours. He underwent detailed diagnostic procedures, and a loop recorder was implanted for continuous long-term monitoring of his heart rhythm. As no cardiac abnormalities were detected upon standard cardiological evaluation, including echocardiography, he was referred to the department of neurology for evaluation of possible epilepsy. Antiseizure medication was started, although brain magnetic resonance imaging (MRI) and electroencephalogram (EEG) recordings were normal. Due to atypical ictal signs and symptoms, the epilepsy treatment was discontinued but subsequently reintroduced after episodes with convulsive movements, tongue biting, and even a shoulder dislocation that occurred following loss of consciousness. However, several episodes recurred despite antiepileptic treatment.

**Fig. 1:**
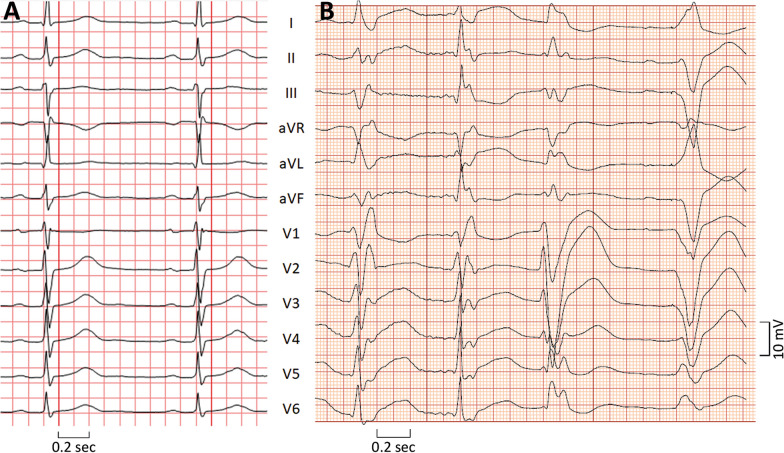
ECG recordings: **a** sinus rhythm with borderline atrioventricular delay, otherwise quite normal—as recorded in between all attacks and **b** gradually subsiding, polymorphic ventricular ectopy was observed after both resuscitations

A total of 8 months after the first episode, the patient was admitted with cardiac arrest. The ambulance team reported polymorphic ventricular tachycardia (VT) degenerating to ventricular fibrillation. Resuscitation, including intubation, was immediately initiated prehospitally. He received multiple direct current cardioversions, but sinus rhythm lasted only a few seconds after each return of spontaneous circulation. Resuscitation was ongoing upon admission to hospital. In addition to advanced cardiac resuscitation, he received amiodarone, magnesium sulfate, and inotropic medical support. Initially, frequent ventricular ectopic beats were observed, with gradual decline in the next hours with a normalized electrocardiogram (Fig. 1). Within the next 2 weeks, the patient recovered completely.

The patient’s troponin T concentration peaked at 7290 ng/L. However, coronary angiography showed no signs of coronary artery disease, which made an ischemic cause unlikely. Repeated echocardiograms and cardiac MRI showed normal biventricular function without any structural, valvular, or vascular abnormalities, or signs of inflammation or scarring. Genetic testing did not identify any genetic variants associated with channelopathies or cardiomyopathies. After 4 weeks in hospital, he underwent implantation of a two-chamber cardiac defibrillator (ICD). The patient was discharged to his home and family, with a scheduled follow-up.

Four weeks after discharge, our patient was readmitted with an identical situation, with the ICD having recorded repeated cardioversions. Again, similar resuscitation procedures terminated the malignant arrhythmias. During ICU monitoring for 2 weeks, he once again recovered with normal cardiac and neurological function. He underwent invasive cardiac electrophysiology testing with supraventricular and ventricular stimulation without provoking any arrhythmias.

The patient had repeated episodes of malignant ventricular arrhythmias without an obvious etiology. These arrhythmias were life-threatening even with appropriate function of the ICD and were accompanied by bizarre neurological symptoms. The patient was instructed to contact the hospital immediately upon recurrence of the symptoms. Several attacks during the coming weeks led to acute admissions with prehospital cardiac monitoring. The patient reported that most episodes started with perioral pain and numbness followed by fatigue and dizziness. Almost every time he was found to have initial hypotension, which resolved in the course of a few hours after initiation of crystalloid infusions.

Due to the combination of symptoms and recurrent polymorphic VT without evidence of structural or functional cardiac abnormalities, nor inherited channelopathies, a toxic agent was finally suspected. A multidisciplinary medical team discussed the case, a toxicologist was consulted, and literature was thoroughly searched. Based on this, aconitine poisoning emerged as a likely culprit. Following another attack of symptoms, serum and urine samples were collected for toxicological analyses, confirming the diagnosis.

Once the cause of the patient’s episodes had been identified, the patient suffered no further attacks. The ICD was explanted after a few months, and he returned to work 6 months after suffering the last cardiac arrest.

### Diagnosis of aconitine poisoning

Following the last symptomatic episode, which occurred shortly after a meal, serum and urine samples were collected for toxicological analyses approximately 5 hours after suspected ingestion. Qualitative analyses by liquid chromatography quadrupole time-of-flight mass spectrometry (LC-Q-TOF-MS) indicated the presence of aconitine in both serum and urine. Quantitative liquid chromatography with tandem mass spectrometry (LC-MS-MS) analyses determined aconitine concentrations in serum and urine of 4.0 ng/mL (6.2 nmol/L) and 87 ng/mL (134 nmol/L), respectively.

**Fig. 2:**
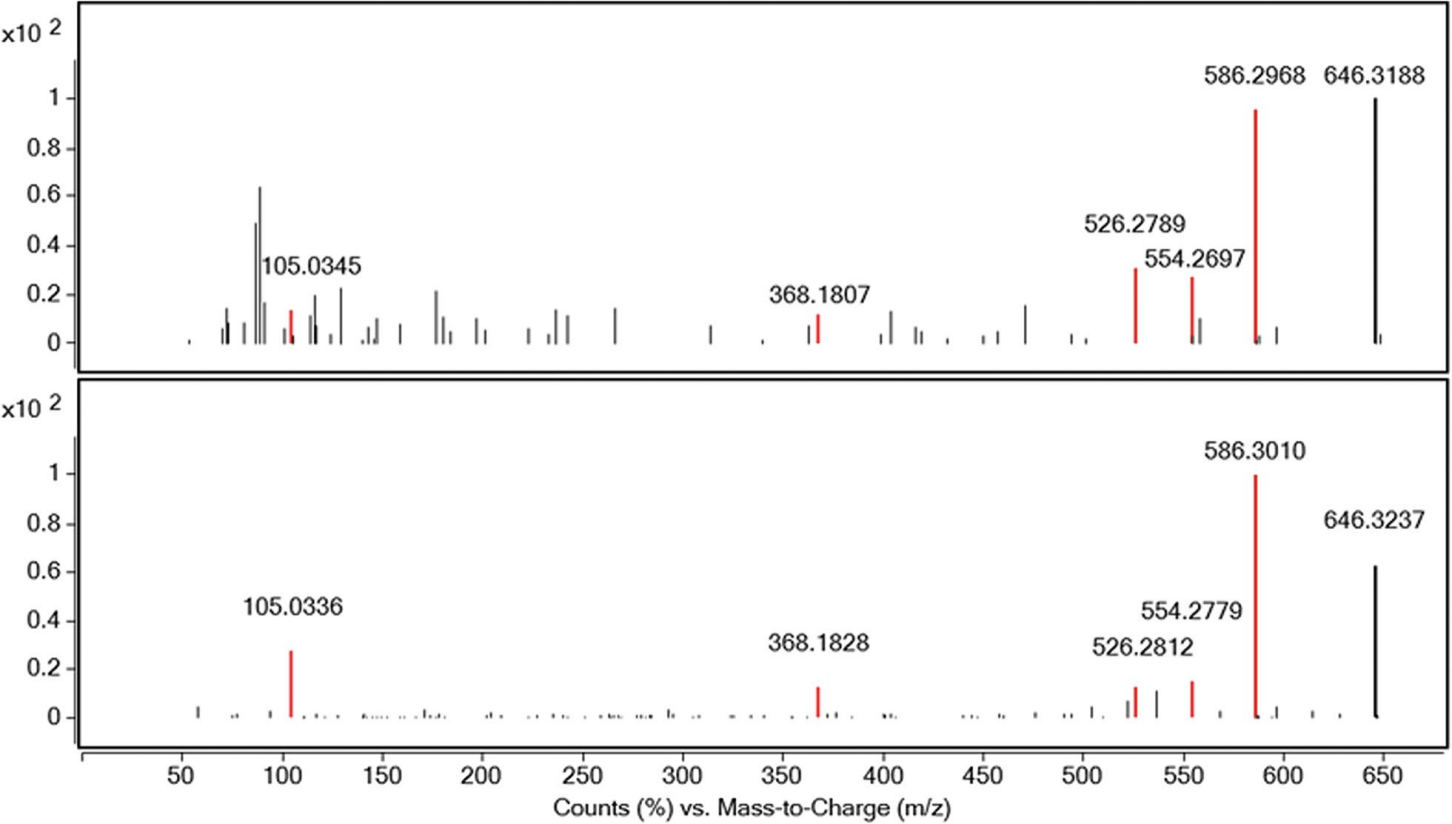
Acquired tandem mass spectrometry (MS/MS) spectrum from analysis by LC-Q-TOF-MS of a hair sample from the patient (upper panel), compared to the full MS/MS spectrum from a certified aconitine reference standard (lower panel). Matching ion fragments are shown in red, with their respective mass-to-charge (*m*/*z*) ratios. The height of the peaks illustrate the relative abundance of ion fragments. The peak of *m*/*z* 646 represents unfragmented aconitine

A hair sample of 3.5 cm was collected 2.5 months after the final admission and was cut into a proximal segment of 1.5 cm and a distal segment of 2 cm. Qualitative analysis by LC-Q-TOF-MS revealed the presence of aconitine in the distal segment (Fig. 2). As hair normally grows at a rate of 1 cm per month, the finding corresponds to the symptomatic period.

Identification by LC-Q-TOF-MS was based on the acquired MS/MS spectrum from analysis of the biological samples, compared to the full MS/MS spectrum from a certified aconitine reference standard (Chiron AS, Trondheim, Norway), as well as matching retention times. The quantification by LC-MS-MS was done in electrospray ionization in positive mode, monitoring aconitine at a mass-to-charge (*m*/*z*) ratio of 646.3 > 526.3 and 646.3 > 368.2. The method’s limit of quantitation was 0.1 ng/mL, and the calibration curve was linear over the 0.1–500 ng/mL range, with a coefficient of determination of > 0.99. Citalopram-d_4_ was used as internal standard. No formal analyses of precision and recovery were undertaken.

We created a clinical scoring system to systematically evaluate the likelihood of aconitine poisoning as reason for the clinical phenotype in each of the admissions (Table 1). This scoring system was based on a review article by Chan, which provided clinical features observed in known cases of aconitine poisoning [3]. We used this scoring system to provide a score for each of the admissions to be considered by the court (Table 2). Only in the last admission was aconitine analyzed and detected in the patient’s serum and urine. Of note, the clinical features presented during this specific admission only gave a score of 2/4. To put this in perspective, a similar score was considered to present a clinical probability of more than 50% for aconitine poisoning in the earlier admissions (except no. 6). If the score was at least 3/4, we considered the probability of aconitine poisoning to be more than 90%, given the fact that aconitine was detected in the last admission (no. 12, Table 2). These translations of scores into crude probabilities were subjective but important to make the quantified assessments that are demanded for the expert statement provided to the court. The scores were provided independently and blinded by two authors (D.J., M.K.S.), and in the only admission with disagreement (no. 1, Table 2), the lowest score was used.

**Table 1:** Clinical features reported in aconitine poisoning classified in four groups; three were organ specific and the fourth contained more unspecific features reported (modified from Chan [3])

	Clinical features reported in aconitine poisoning	Frequency (%)
1	Neurological (N; perioral numbness and paresthesias, convulsions, and reduced muscular strength)	94–100
2	Cardiovascular [C; arrhythmias, hypotension (< 90 mmHg systolic), chest pain, and palpitations]	78–83
3	Gastrointestinal (G; abdominal pain, nausea, vomiting, and diarrhea)	53–72
4	Other (O; dizziness, fatigue, sweating, hyperventilation, lacrimation, and headache)	33–77

A possible mnemonic here could be cardiovascular, other, neurological, and gastrointestinal features score (CONG score)

**Table 2:** Aconitine score in 12 previous hospital admissions thought to be associated with aconitine exposure

Admission no.	Aconitine score	Clinical features present	Comments
1	3/4^a^	N, C, G, (O)	
2	3/4	N, C, G	
3	3/4	N, C, G	
4	2/4	N, G	
5	2/4	N, G	
6	–	Shoulder fracture caused by convulsions	May be indirectly related
7	3/4	N, C, G	Cardiac arrest
8	3/4	N, C, G	
9	2/4	N, C	Cardiac arrest
10	2/4	C, G	
11	2/4	C, G	
12	2/4	N, C	Aconitine detected

Admission no. 6 was not scored

^a^One rater had 4/4 here but then the lowest score was used.

*N* neurological, *C* cardiovascular, *G* gastrointestinal, *O* other

## Discussion

The patient had lived through a decade of repeated medical emergencies involving various organ systems. Despite seemingly appropriate investigations, the etiologies had remained obscure. After a series of admissions with the same combination of unusual clinical features, poisoning was finally suspected. In retrospect, the history clearly suggests that the same toxic agent was the cause of all unexplained emergency events throughout this period of 12 months. However, a toxic agent explaining the earlier episodes of renal and hepatic failure has not been identified. Of note, poisonings with *Cortinarius* species and *Amanita virosa*, respectively, might well have explained these episodes.

Systematic ruling out of structural or primary electrical heart disease, as well as neurological disorders, was important in our case. In such cases, poisoning should be part of the diagnostic possibilities. For our patient, the mystery was finally solved when the likely culprit was suspected due to the peculiar and stereotypical development of clinical features during each episode starting in a similar manner, that is, perioral paresthesias and numbness along with abdominal pain, nausea, and vomiting. He also felt fatigue and dizziness. Several of the attacks culminated in syncope and convulsions, sometimes with trauma and even tongue biting. Eyewitness reports were probably misleading, but two attacks proceeded to documented cardiac arrest with malignant ventricular arrhythmias. All of these symptoms and findings were characteristic for poisoning with aconitine [2].

The pathophysiology of aconitine poisoning is well in line with its pharmacodynamics: a strong affinity to binding site 2 of the α subunit of voltage-gated sodium channels, partly activating the channels and leading to sustained inward sodium flow [3, 13]. In addition, aconitine binds to potassium channels in cardiomyocytes and might even affect voltage-dependent calcium channels, although the latter might be species dependent [14–16]. The resulting prolongation of the plateau phase of the cardiac action potential increases the risk of early afterdepolarizations, as well as sodium accumulation with secondary calcium overload and delayed afterdepolarizations [14]. The result is an increased propensity for conduction defects that might lead to bradycardias, as well as ectopic atrial and ventricular activation. All of these manifestations were seen in our patient, in addition to polymorphic VT. Importantly, the corrected QT (QT_c_) interval was within the normal range, and the resting ECG had no other signs of channelopathies or cardiomyopathies. Negative results from genetic testing and electrophysiological testing further reduced the probability of a constitutional propensity for arrhythmias.

The other dominant features experienced by our patient might be due to activation of the muscarinic receptors, which contributes to hypotension and bradyarrhythmia, while increased refractoriness of neurons and skeletal muscle leads to paresthesia in the mouth and limbs as well as muscle weakness [10].

Regarding treatment of short-lasting (few hours) arrhythmias and circulatory failure in aconitine poisoning, we suggest prolonged cardiocompression with mechanical devices, circulatory support [extracorporeal membrane oxygenation (ECMO)], and temporary pacing, which we also used successfully in another case, rather than antiarrhythmics.

The present case also illustrates problematic aspects of covert poisonings. The patient experienced a decade of different severe conditions with a surprisingly rapid recovery. Encountering different medical disciplines and departments, along with deceptive information from the offender, probably hampered the diagnostic process. The case serves as a reminder to emergency care personnel and clinicians evaluating ambiguous cases to consider covert poisoning as a potential explanation, even in the absence of an obvious psychosocial setting suggesting malevolent behavior. If poisoning is suspected, the case should be discussed with a clinical toxicologist or a poison information center, and biological samples should be secured.

The breakthrough in this particular case came after extensive multidisciplinary evaluation and discussions. Of importance for this team-based evaluation was the fact that the patient was admitted to the same department when new attacks occurred. The diagnosis was made after a ruleout of other potential explanations, a recurrent, yet transient, clinical presentation, and confirmation of a suspected toxic agent by specific analyses in blood and urine secured during a bout of clinical features.

An aconitine concentration of 4.0 ng/mL was measured in serum approximately 5 hours after the likely time of intake. Assuming a time to peak plasma concentration (Tmax) of 1 hour and an elimination half-life of 3–4 hours, the aconitine concentration in serum most probably reached the maximum concentration after administration (Cmax) of at least 8 ng/mL during the first hour after intake [9]. Lower concentrations have been associated with toxic effects on the cardiovascular, nervous, and gastrointestinal system in other case reports [9, 17, 18]. In most reported deaths from aconitine poisoning, aconitine concentrations in blood have exceeded 10 ng/mL, although concentrations as low as 3.6 ng/mL may be considered lethal unless treated [19].

In aconitine poisoning, concentrations have been reported to be considerably higher in urine than in blood [17, 18], which was also the case in our patient. This suggests that aconitine is detectable for a longer time after intake in urine than in blood and underlines the importance of securing both blood and urine samples from the patient, especially if the possibility of aconitine poisoning comes to mind after the acute symptomatic period has passed.

We were also able to detect aconitine in a hair sample, which was collected about 2.5 months after the final attack. Aconitine was present in the segment of hair that grew approximately 1.5–3.5 cm from the scalp, which corresponded to a period during which the patient probably suffered several episodes of aconitine poisoning. Thus, hair analysis could be a diagnostic possibility in cases where a suspicion of aconitine poisoning arises in retrospect, too late for the substance to be detected in body fluids, as has also been shown for arsenic poisoning [20].

The proposed aconitine score was introduced as a retrospective scoring system for the court trial to evaluate the probability of aconitine poisoning in the previous admissions with similar clinical features, from which there were no biological samples available to confirm the diagnosis by aconitine analyses (Table 2). The court’s conclusion was that all admissions in Table 2 were caused by aconitine. This score may be useful in similar situations where diagnoses in retrospect are sought—both in clinical or forensic settings—based on a confirmed diagnosis of aconitine poisoning (urine, serum, and hair analyses), as in this patient’s 12th admission. The score has, however, not been validated for prospective diagnosis of aconitine poisoning.

## Conclusion

Aconitine poisoning is a potentially life-threatening situation, which may represent a substantial diagnostic challenge when the exposure is obscure. A detailed history revealing the characteristic cascade of clinical features starting with perioral sensations and gastrointestinal manifestations followed by cardiac arrhythmias and syncope, should lead to the clinical suspicion. Detection of aconitine in body fluids is diagnostic; in the immediate aftermath of signs and symptoms aconitine may still be present in urine and also later in hair. We offer a clinical score that can be applied retrospectively to events that may be consistent with aconitine poisoning.

### Addendum

After confirmation of aconitine poisoning, the patient’s wife of more than 10 years, who had appeared concerned and supportive, was suspected of having extracted aconitine from garden plants (*Aconitum napellus*) for administration in the patient’s food and beverages. After two trials, she was sentenced in the court of appeal to 11 years in prison for attempted murder.

## Data Availability

Data sharing is not applicable to this article as no data sets were generated or analyzed during the current work.
